# Causes of hypercalcemia in renal transplant recipients: persistent hyperparathyroidism and others

**DOI:** 10.1590/1414-431X202010558

**Published:** 2021-04-26

**Authors:** M. Moyses-Neto, T.M.P. Garcia, M.E.P. Nardin, V.A. Muglia, C.A.F. Molina, E.A. Romao

**Affiliations:** 1 Divisão de Nefrologia, Departamento de Clinica Médica, Faculdade de Medicina de Ribeirão Preto, Universidade de São Paulo, Ribeirão Preto, SP, Brasil; 2 Divisão de Urologia, Departamento de Cirurgia, Faculdade de Medicina de Ribeirão Preto, Universidade de São Paulo, Ribeirão Preto, SP, Brasil

**Keywords:** Hypercalcemia, Kidney transplant, Hyperparathyroidism, Granulomatous infections, Lymphoma

## Abstract

Hypercalcemia is common in patients after kidney transplantation (KTx) and is associated with persistent hyperparathyroidism in the majority of cases. This retrospective, single-center study evaluated the prevalence of hypercalcemia after KTx. KTx recipients were evaluated for 7 years after receiving kidneys from living or deceased donors. A total of 301 patients were evaluated; 67 patients had hypercalcemia at some point during the follow-up period. The median follow-up time for all 67 patients was 62 months (44; 80). Overall, 45 cases of hypercalcemia were classified as related to persistent post-transplant hyperparathyroidism (group A), 16 were classified as “transient post-transplant hypercalcemia” (group B), and 3 had causes secondary to other diseases (1 related to tuberculosis, 1 related to histoplasmosis, and 1 related to lymphoma). The other 3 patients had hypercalcemia of unknown etiology, which is still under investigation. In group A, the onset of hypercalcemia after KTx was not significantly different from that of the other groups, but the median duration of hypercalcemia in group A was 25 months (12.5; 53), longer than in group B, where the median duration of hypercalcemia was only 12 months (10; 15) (P<0.002). The median parathyroid hormone blood levels around 12 months after KTx were 210 pg/mL (141; 352) in group A and 72.5 pg/mL (54; 95) in group B (P<0.0001). Hypercalcemia post-KTx is not infrequent and its prevalence in this center was 22.2%. Persistent hyperparathyroidism was the most frequent cause, but other important etiologies must not be forgotten, especially granulomatous diseases and malignancies.

## Introduction

A significant number of kidney transplant patients have parathyroid hormone (PTH) levels that decline substantially during the first 6 months after kidney transplantation (KTx). However, persistent hyperparathyroidism in KTx recipients with adequate allograft function still results in high PTH levels (1,2). Persistent hyperparathyroidism can still be observed in 30-60% of these patients one year after KTx (3–6). Most often, the elevated PTH level in this later period is responsible for an increase in serum calcium. The prevalence of hypercalcemia in two recent studies was around 15% and was always associated with PTH disturbances (6,7). Increased serum calcium may also occur in association with low PTH levels. In this case, other causes, such as malignancy and opportunistic infection, should be considered. It could be related to cancer, multiple myeloma, lymphomas, sarcoidosis, and infectious granulomatous diseases, such as tuberculosis or fungal infections. Hypercalcemia in conjunction with *Pneumocystis jirovecii* pneumonia (PCP) is being reported more frequently in immunocompromised patients (8). The aim of the current study was to evaluate the prevalence of hypercalcemia and its causes or associations in KTx patients.

## Material and Methods

This was a retrospective study conducted through the analysis of medical records, which was carried out at the Kidney Transplantation Unit of a University Hospital. Kidney transplant recipients were evaluated from 2010 to 2016 (7 years) after receiving kidneys from living or deceased donors and followed-up until the end of 2018. All kidney transplant recipients over 18 years of age who were transplanted in this hospital and were followed-up at the Kidney Transplant Clinic at this University Hospital were included. Patients who had early graft loss (in the first 3 months), died, were transferred to other services, or had inadequate information in the medical records were excluded. Patients who had hypercalcemia after KTx and who maintained this change for at least 3 consecutive months were tracked by the hospital system. After this verification for 3 months, serum calcium was checked according to routine blood samples from the Service: every month in the first year after KTx, every 2 months in the second year, and every 3 months thereafter. Hypercalcemia was defined as blood calcium levels above 10.5 mg/dL (normal range: 8.5-10.5 mg/dL). The highest blood calcium level reached by the patients during the observation period was also verified. Ionized calcium was not checked because it was not within the Service's routine protocol. Although not the main aim of this study, phosphorus levels in the hypercalcemic patients were also tracked during the follow-up (normal levels: 2.5-5.6 mg/dL). Persistent hyperparathyroidism was considered for patients who maintained increased intact PTH levels after 12 months from Ktx (normal range: 14.5-87.1 pg/mL, chemiluminescence assay). The blood levels of PTH and blood creatinine were measured about 12 months after KTx. Blood PTH levels of patients prior to KTx were also checked. We also registered the time of occurrence, duration of hypercalcemia, and hypophosphatemia blood levels (<2.5 mg/dL). Regarding the KTx hypercalcemic patients, we also collected the following information: sex, age, types of dialysis prior to KTx, and induction and maintenance of immunosuppression.

Groups were compared using the non-parametric Mann-Whitney test. In all cases, P<0.05 was considered statistically significant. The data are reported as median and percentile (25th; 75th) values. The study was approved by the hospital's Research Ethics Committee.

## Results

During the 7-year period, 400 patients received transplants. Ninety-nine patients were excluded for early graft loss, death, or transfer to other services. A total of 301 patients were evaluated, 67 (22.2%) of whom had hypercalcemia at some point during the study period. Of these 67 patients, 89.5% received kidneys from deceased donors and 10.5% received kidneys from living donors. Thirty-five patients (52.2%) were female, the mean age of all patients was 48.1±10.9 years, the median time of dialysis pre-transplant for all 67 patients was 62 months (45; 90), and the most common known causes of their end stage renal disease were glomerulopathies, diabetes, and polycystic kidney disease. Prior to transplantation, 58 patients (86.5%) underwent hemodialysis, 5 (7.4%) underwent both hemodialysis and peritoneal dialysis, 2 underwent peritoneal dialysis (2.9%), and data were not available for 2 (2.9%) patients. Most of the patients had received induction with ATG (thymoglobulin) (56.7%), and the most frequently used drugs for maintenance immunosuppression were prednisone, mycophenolate, and tacrolimus (78.0% of the patients). The median follow-up time for all 67 patients was 62 months (44; 80) and onset of hypercalcemia was 2 months (1; 7). The median duration of hypercalcemia was 18 months (11; 42). Of the 67 patients studied, 45 (67.1%) cases of hypercalcemia were classified as related to persistent post-transplant hyperparathyroidism (group A) and 16 patients (23.8%) were classified as transient post-transplant hypercalcemia (group B) with decreased calcemia and PTH to almost normal levels around the first year after KTx.

In the 45 patients in group A, the onset of hypercalcemia occurred in the first months with a median of 2 months (1; 6.5), which was similar to the onset of hypercalcemia in the 16 patients of group B, who had a median onset of 2.5 months (1; 5). The median duration of hypercalcemia was 25 months (12.5; 53) in group A and 12 months (10; 15) in group B, with a significant difference between groups (P<0.002). In group A, the median highest blood calcium level was 12.2 mg/dL (11.8; 12.6), whereas it was 11.6 mg/dL (11.3; 11.8) in group B. There was a significant difference in the highest blood calcium values between groups A and B (P<0.001) (Table 1).

**Table 1:** Blood calcium, phosphorus, parathyroid hormone (PTH), and blood creatinine data from patients with hypercalcemia after kidney transplantation (KTx) (groups A and B).

	Group A (45 patients)	Group B (16 patients)	P
Highest blood calcium (mg/dL)	12.2 (11.8; 12.6)	11.6 (11.3; 11.8)	<0.001
Onset of hypercalcemia (months)	2.0 (1; 6.5)	2.5 (1; 5)	NS
Duration of hypercalcemia (months)	25.0 (12.5; 53)	12.0 (10; 15)	<0.002
Blood phosphorus (mg/dL)	2.0 (2.0; 2.0)	2.0 (1.0; 2.0)	NS
Duration of hypophosphatemia (months)	3.0 (2.0; 8.0)	1.5 (1.0; 2.0)	<0.003
PTH levels around 12 months after KTx (pg/mL)	210 (141; 352)^a^	72.5 (54; 95)	<0.0001
Blood creatinine 12 months after KTx (mg/dL)	1.3 (1.1; 1.6)	1.2 (1.0; 1.4)	NS

Data are reported as median and percentiles (25th; 75th). The Mann-Whitney test was used for statistical analyses. NS: not significant. Group A: persistent hyperparathyroidism; Group B: transient hypercalcemia. ^a^In this group, only 44 patients had PTH level data available.

In the other 6 patients, 3 had causes secondary to other diseases: one case was related to tuberculosis, one to histoplasmosis, and one to lymphoma. The other 3 patients had hypercalcemia of unknown etiology. In group A, hypercalcemia was usually handled with cinacalcet (48.8%), cinacalcet and vitamin D (28.8%), or vitamin D3 and/or bisphosphonates (23.3%). Even with these medications and treatment with one or more drugs, parathyroidectomy was performed in 11 patients (24.4%) due to the lack of response to those medications in the study period. In group B, there was no need for specific treatment.

In the cases without apparent etiology, the causes are still under investigation. In these 3 patients, PTH levels were within the normal range (one case) or slightly above the normal range (two cases) at follow-up, probably due to the persistence of hyperparathyroidism.

Blood PTH levels were different in groups A and B around the first 12 months after KTx. In group A, blood samples were collected at a median follow-up time of 12 months (9.2; 12.0) after KTx, with median blood PTH levels of 210 pg/mL (141; 352). In group B, the blood PTH levels were evaluated at a median follow-up time of 8 months (7.0; 13.7) with median blood PTH levels of 72.5 pg/mL (54.5; 95.2) (Table 1). There was a significant difference in the blood PTH levels between groups A and B (P<0.0001). No significant difference between the groups in terms of the time of blood collection (P>0.05) was found. Blood creatinine one year after KTx had a median of 1.3 mg/dL (1.1; 1.6) in group A and 1.2 mg/dL (1.0; 1.4) in group B and the results were not different between groups A and B (P>0.05) (Table 1). Thirty-six patients of 45 in group A had data about blood levels of PTH before KTx with a median of 645 pg/dL (290; 1167). In group B, 8 patients of 16 had these results showing median blood levels of PTH before KTx of 143 pg/dL (70; 345) with a significant difference between groups A and B (P<0.003).

Blood phosphorus levels were also evaluated in the two main groups. In the 45 patients in group A, 32 patients (71%) had blood phosphorus levels below 2.5 mg/dL (normal value: 2.5-5.6 mg/dL) with a median value of 2.0 mg/dL (2; 2), whereas in group B, 8 patients (50%) presented phosphorus levels below 2.5 mg/dL, with a median value of 2.0 mg/dL (1.0; 2.0). There was no significant difference in terms of hypophosphatemia between groups A and B. As for the duration of hypophosphatemia, the median duration was 3 months (2.0; 8.0) in group A and 1.5 months (1.0; 2.0) in group B, with a significant difference between groups (P<0.003) (Table 1). We generally considered serum phosphate levels of 1.5 mg/dL or less as suggestive for considering supplementation therapy as symptoms like muscle weakness could be present.

## Discussion

We performed a retrospective observational study of 301 patients who underwent KTx between 2010 and 2016 and all patients with functioning renal allografts were followed-up until the end of 2018. The prevalence of hypercalcemia found in this study was 22.2%. Hypercalcemia post-KTx is a common finding and in the majority of cases is related to persistent hyperparathyroidism after KTx. In this study, we made an interesting observation that allowed us to recognize and classify some different categories for hypercalcemia after renal transplantation. In the first and more prevalent category, we had 45 patients with persistent hyperparathyroidism (group A) and in the second category, we had 16 patients who presented with what we named “transient post-transplant hypercalcemia” (group B). Another important category that we should always pay attention to are the patients with hypercalcemia due to other associated diseases, like granulomatous diseases or neoplasms (3 cases).

Other associated diseases that provoke hypercalcemia are sarcoidosis and many other granulomatous diseases, like histoplasmosis, tuberculosis, leprosy, pneumocystis pneumonia, and vitamin D toxicity (8–11). Therefore, it is important to be aware of such conditions because their treatments must be very specific. In this study, three patients who presented with post-KTx hypercalcemia had associated diseases: two had infectious granulomatous diseases (one case of tuberculosis and one case of pulmonary histoplasmosis) and one patient had lymphoma. The tuberculosis case was a female with cutaneous and articular infections in the first year post-KTx and hypercalcemia that started 8 months after KTx. This fact was unusual since the median onset of hypercalcemia in most of the patients in both groups was 2 months. The diagnosis of tuberculosis was made some months after KTx (PTH levels were already normal). She received treatment and her infection was cured, but one year after treatment, she lost her graft and returned to dialysis. The other patient with granulomatous disease was a female patient who presented a pulmonary granuloma due to histoplasmosis. The hypercalcemia started 10 months after surgery and her PTH levels were in the normal range. She was treated with antifungal medications and responded to treatment. The third patient was a man who had hypercalcemia 7 months post-surgery and also presented with hepatomegaly. In this specific case, his PTH blood levels were also above the normal range at the time of diagnosis. A liver biopsy showed post-transplant lymphoproliferative disease (PTLD), a diffuse large B-cell lymphoma. He received chemotherapy and responded to the tumor treatment.

PTH levels decrease significantly during the first 3 months after transplantation, but typically stabilize at normal or elevated values after 1 year. In some patients, especially those with mild disease before the transplant, secondary hyperparathyroidism resolves after KTx as a more normal glomerular filtration rate is restored (12). However, persistent hyperparathyroidism is reported to occur in approximately 15 to 50% of patients following transplantation (13). This is because of the persistence of structural changes in the parathyroid glands, such as hyperplasia and adenoma formation, despite removal of the initial stimuli for hyperparathyroidism (6,14). Low calcium levels before transplantation tend to increase after transplantation and stabilize at the higher end of the normal range within 6 months (12). In group A (persistent hyperparathyroidism) patients, hypercalcemia occurred at higher levels and for longer durations than in group B (transient hypercalcemia).

Regarding PTH levels before KTx, the majority of patients in group A showed high levels of PTH (pre-transplant hyperparathyroidism) compared with group B. Some authors also show that pre-transplant hyperparathyroidism is correlated with persistent hyperparathyroidism and hypercalcemia post-KTx (7). It is generally believed that post-transplant hyperparathyroidism may reflect a degree of decreased renal function, but one year after KTx blood levels of creatinine were not different in the groups.

In patients of group A, persistent hyperparathyroidism was usually handled with medications, but 11 patients did not respond in the study period and parathyroidectomy was performed in these patients. Tertiary hyperparathyroidism following KTx is relatively common and increases the long-term risk of death-censored graft failure. This finding highlights the need for better management of this disturbance and finding more appropriate timing to perform parathyroidectomy (14). In this group, blood PTH levels tended to increase around 12 months after KTx; in contrast, in group B, the blood PTH levels tended to decrease around the same time.

Hypercalcemia started at the same time in both groups of patients; however, in group B, the median hypercalcemia duration was lower than that of group A. This kind of transient post-transplant hypercalcemia was likely due to improved parathyroid function due to the recovery of normal circulating calcitriol levels, which is observed during the first months after KTx as renal function starts to improve (15).

Hypophosphatemia is very common after KTx and has been reported in 40-90% of patients in the early period following transplantation (16). KTx induces specific changes in phosphate metabolism. Many factors are recognized as contributing to hypophosphatemia, mainly persistent high levels of FGF-23 and PTH and the influence of immunosuppressive drugs (17). Some authors showed that phosphate levels rapidly decrease after transplantation, but this tended to resolve itself within 2 months (11). Due to phosphaturia, phosphate mobilization occurs from the bone and there is an increase in intestinal absorption of 1,25-dihydroxyvitamin D3; this mechanism helps with the normalization of phosphaturia and phosphatemia (17). In the present study, the prevalence of hypophosphatemia was 71% in group A and 50% in group B. Some authors associate hypophosphatemia development with a lower risk of graft failure, compared to cases who did not develop hypophosphatemia (18). Phosphate supplementation may contribute to calcification of the transplanted kidney, and thus these supplements should be prescribed with the minimum dose possible (17).

The aim of the study was not to evaluate the results of possible treatments for hypercalcemia or hyperparathyroidism, but the approach to manage hypercalcemia depends on the blood calcium levels and the presence or absence of clinical manifestations. Most patients with mild hypercalcemia (<12.0 mg/dL) are asymptomatic and do not require acute treatment. Patients with moderate elevations in serum calcium (12.0-14.0 mg/dL) may develop symptoms or have worse kidney function when levels rise rapidly; these symptomatic patients require immediate intervention. All patients with serum calcium levels >14.0 mg/dL require aggressive treatment. In this case, adequate fluid intake, furosemide, and sometimes bisphosphonates and the avoidance of thiazide diuretics, calcium, and vitamin D supplements are encouraged. Severe hypercalcemia can cause acute kidney injury in the allograft due to volume contraction and by reducing perfusion to the allograft by direct vasoconstriction (9 [Bibr B10],^19^,^20^).

Hypercalcemia post-KTx is not infrequent and its prevalence in this center was 22.2%. Persistent hyperparathyroidism was the most frequent cause and was responsible for eleven cases of parathyroidectomy in this study period. Other important causes of hypercalcemia must not be forgotten, especially infectious granulomatous diseases and malignancies that will need specific treatment.
